# A Review of the Unintentional Release of Feral Genetically Modified Rapeseed into the Environment

**DOI:** 10.3390/biology10121264

**Published:** 2021-12-03

**Authors:** Soo-In Sohn, Subramani Pandian, Young-Ju Oh, Hyeon-Jung Kang, Tae-Hun Ryu, Woo-Suk Cho, Eun-Kyoung Shin, Kong-Sik Shin

**Affiliations:** 1 Department of Agricultural Biotechnology, National Institute of Agricultural Sciences, Rural Development Administration, Jeonju 54874, Korea; pandiannsp7@gmail.com (S.P.); happykorean@korea.kr (H.-J.K.); thryu@korea.kr (T.-H.R.); phyto@korea.kr (W.-S.C.); novis7@korea.kr (E.-K.S.); 2 Institute for Future Environmental Ecology Co., Ltd., Jeonju 54883, Korea; 50joo@hanmail.net; 3 Audit and Inspection Office, Rural Development Administration, Jeonju 54875, Korea; kongsiks@korea.kr

**Keywords:** genetically modified crops, management, feral populations, unintentional release, herbicide resistance, environmental safety, *Brassica napus*, rapeseed

## Abstract

**Simple Summary:**

With the advent of genetic engineering technology, the development and cultivation of genetically modified (GM) crops has increased. They were mainly developed for high yielding, herbicide resistance, and tolerance against different biotic and abiotic stresses. Rapeseed, also known as canola, was developed mainly for herbicide resistance and to increase the production of canola oil. Since it forms weedy, feral populations and has a proven ability to hybridize with its close relatives, it is important to manage the GM crops’ cultivation and spread, especially the rapeseed. Several studies have reported that the spread of GM rapeseed in non-GM fields and road verges is possible due to transport and agronomic practices, and it may become a weed. Hence, in this review, we summarized the cases of unintentional spread of feral GM rapeseed in the fields and road verges. In addition, we made recommendations for the effective management of feral GM and non-GM rapeseed in agricultural fields and along roads.

**Abstract:**

Globally, the cultivation area of genetically modified (GM) crops is increasing dramatically. Despite their well-known benefits, they may also pose many risks to agriculture and the environment. Among the various GM crops, GM rapeseed (*Brassica napus* L.) is widely cultivated, mainly for oil production. At the same time, *B. napus* possesses a number of characteristics, including the ability to form feral populations and act as small-seeded weeds, and has a high potential for hybridization with other species. In this review, we provide an overview of the commercialization, approval status, and cultivation of GM rapeseed, as well as the status of the feral rapeseed populations. In addition, we highlight the case studies on the unintentional environmental release of GM rapeseed during transportation in several countries. Previous studies suggest that the main reason for the unintentional release is seed spillage during transport/importing of rapeseed in both GM rapeseed-cultivating and -non-cultivating countries. Despite the fact that incidents of unintentional release have been recorded often, there have been no reports of serious detrimental consequences. However, since rapeseed has a high potential for hybridization, the possibilities of gene flow within the genus, especially with *B. rapa*, are relatively significant, and considering their weedy properties, effective management methods are needed. Hence, we recommend that specific programs be used for the effective monitoring of environmental releases of GM rapeseed as well as management to avoid environmental and agricultural perturbations.

## 1. Introduction

**Figure 1:**
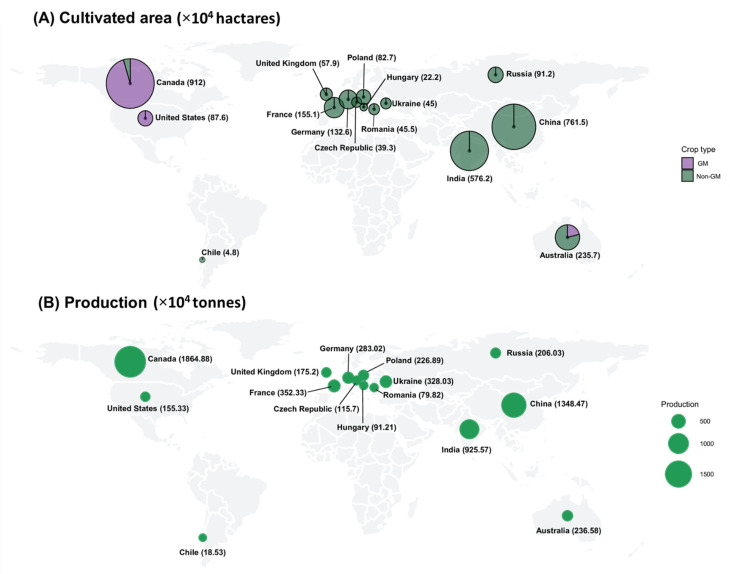
Production and cultivation area of rapeseed in major cultivating countries (**A**). Cultivation area of rapeseed (GM and non-GM) (in hectares); (**B**). Production of rapeseed (in tonnes). Source: FDA Statistics (https://www.fao.org/faostat/en/#data; accessed on 25 September 2021).

Rapeseed (*Brassica napus* L., AACC, 2n = 38) also known as canola, belongs to the *Brassicaceae* family, which contains 338 genera and 3709 species [1]. It is one of the most economically important oilseed crops worldwide, with an annual yield of 75 million tonnes [2] (Figure 1; Table 1). Since rapeseed is closely related to many weeds and wild species, it has a high degree of outcrossing (20–40%), generates a large amount of pollen and has favorable conditions for gene transfer. Several investigations have shown that *B. juncea*, *B. rapa*, *Hirschfeldia incana*, *Sinapis arvensis*, and *Raphanus raphanistrum* are capable of hybridization with *B. napus* [3,4,5]. The extent of outcrossing is determined by the breed, local topography, environmental conditions, and insect pollinator availability [6]. The potential of pollen-mediated hybridization of rapeseed is comparable with that of rice, sugar beet, and sunflower, for several reasons [7]. In general, it is highly pollinated by wind and insects, especially honey bees [8]. Pollinators such as bees and other insects can travel up to several kilometers [9]. *B. napus* has a wide range of pollen distribution due to its small pollen size. The majority of the pollen is deposited within a 100-m radius of the pollen source. Despite the fact that the crossing rate drops dramatically between 10 and 50 m from the pollen source [10,11], a low frequency of cross-pollination has also been recorded even at a distance of 4 km from the source [12,13].

Generally, rapeseed can remain in the soil for a long time and thus contribute to seed banks and originate volunteer populations in subsequent years [14]. Volunteer is a weed in agricultural systems, since it competes with crops for water, nutrients, and sunlight, subsequently reducing production [15]. Volunteers can be found in seeds deposited by improperly cleaned farm machinery, seed transfers from adjacent fields, and seeds spilled from existing seed banks and transport vehicles [16,17,18,19]. The density of annual volunteers is highest in the first year after planting, and it decreases by up to 99% in the second year after planting [20]. In some cases, volunteer is the most prevalent weed species after 1–3 years of cultivation of rapeseed. It has been reported that the seeds of rapeseed can survive for 4–15 years in the land without germinating [20,21]. The average seed loss during harvesting is 5%, or around 2000 to 3600 seeds per square meter [22]. The small seed size of rapeseed results in considerable seed bank additions, despite the low ratio of yield losses [23]. The persistence and quantity of rapeseed volunteers in subsequent crops is also affected by seed dormancy [24], which prevents intact, viable seeds from germinating under favorable conditions. There are two types of seed dormancy: primary dormancy and secondary dormancy [25]. Primary dormancy is a phenomenon that prevents seed germination during the maturation process as well as for a period of time after the seed is removed from the parent plant [25,26]. After maturation, a period is required for seeds to germinate by breaking the primary dormancy [26,27]. Secondary dormancy is defined as a decrease in germination that occurs after seeds are separated from the parent plant [25,26] in response to certain environmental conditions, such as seed burial, large temperature fluctuations, prolonged darkness, osmotic conditions, and limited oxygen [26,28]. A significant proportion of seeds can develop secondary dormancy, allowing them to persist and survive for several years in the soil, thus generating seed bank populations. 

**Figure 2:**
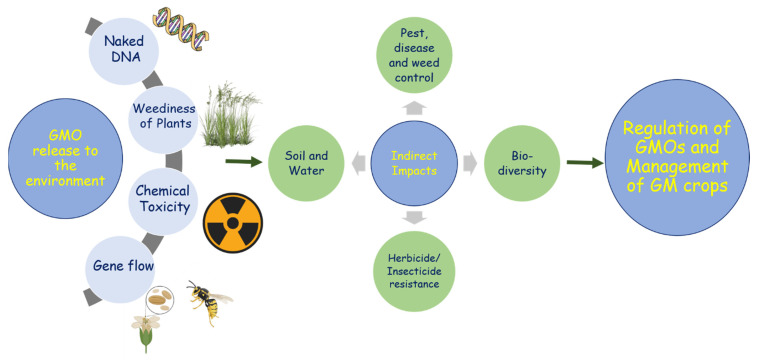
General overview of the environmental release of GMOs and their impacts on agriculture and the environment.

Ever since the first commercialization of genetically modified (GM) crops, in 1996, GM rapeseed cultivars developed for glyphosate and glufosinate herbicide tolerance have escaped cultivation. Since then, there has been a widespread escape and survival of transgenic rapeseed on Canadian roadsides [17,29,30]. Since these reports, wild rapeseed populations containing a proportion of GM plants have been reported in the United States, the United Kingdom, France, Australia, Switzerland, Austria, Sweden, and Japan [31,32]. Among these countries, Canada, the United States, and Australia grow transgenic rapeseed, while the United Kingdom, France, Austria, Sweden, and Japan import but do not grow it, and Switzerland neither cultivates nor imports it. The spread of transgenic rapeseed was witnessed in these nations irrespective of whether transgenic rapeseed was imported and/or farmed. Nowadays, the adverse agricultural and environmental impacts (Figure 2) associated with genetic modification and changes in agricultural practices are hotly debated. Farmers have embraced transgenic rapeseed for its operational benefits, but the coexistence of transformants and non-transformants poses a risk of the inserted transgene escaping [33,34]. In general, there are no previous reports on the environmental impacts of GM rapeseed with modified oil composition compared with non-GM rapeseed. However, while cultivating GM herbicide-resistant (GMHT) rapeseed, the intense use of herbicides for management practices leads to environmental perturbations and loss of biodiversity and may develop herbicide resistance in random crops by gene flow (Figure 2) [7,22,31]. For a mutual coexistence, understanding the process of GM plants and the resulting transgene growing outside the cultivation and spreading to adjacent non-wild habitats is crucial, and appropriate management measures should also be established accordingly. As a response, in this review, we would like to discuss the different types of GM rapeseed that have been commercialized during the last 25 years, the status of approval by country, and cases of transgene escape and their management measures.

## 2. Commercialization of GM Rapeseed

Initially, four types of GM rapeseed have been developed for commercialization, including glyphosate, glufosinate resistance, fatty acid composition modification, and male infertility plants. Different GM rapeseed events, genes, and their properties are provided in Table S1. Glyphosate resistance can be conferred by two genes. Among them, CP4-EPSPS is derived from *Agrobacterium* CP4, which encodes 5-enolpyruvylshikimate-3-phosphate synthase, an herbicide-insensitive enzyme that is a glyphosate target enzyme [35]. The other one is glyphosate oxidoreductase [36], which is an enzyme that degrades glyphosate. Glufosinate resistance is conferred by a single bar gene encoding phosphinothricin-N-acetyl transferase (PAT), an enzyme that inactivates glufosinate [37]. Furthermore, the Barnase gene isolated from *Bacillus amyloliquefaciens* [38] encodes a ribonuclease that is only produced in the tapetum cells of the pollen sac during anther development and is controlled by a tapetum-specific promoter. Generally, male infertility is caused by the Barnase gene, which alters the RNA production, disrupts the normal cellular activity, and prevents the early anther development. The barstar gene, obtained from *B. amyloliquefaciens*, can help cure male infertility [39]. The Barnase ribonuclease is inhibited by the Barstar gene, which encodes a ribonuclease inhibitor. Thus, a Barnase/Barstar hybrid line that can develop normal anthers and restore fertility can be produced by the pollinator system.

Countries that produce or import GM rapeseed are approved/supervised according to whether the seeds are used for food, feed, or cultivation. Since the first herbicide-tolerant transgenic canola was certified for commercial cultivation in Canada, in 1995, it has been permitted for food, feed, and agriculture in 15 countries. Among these countries, Canada, the United States, Australia, Japan, and Chile have approved GM rapeseed for production, and it is currently grown in four countries, excluding Japan. Even though GM rapeseed is not produced in other countries, it has been discovered that it is unintentionally released into the environment due to problems occurring at the unloading site during the importation of GM rapeseed [40,41].

## 3. Cases for the Unintentional Release of GM Rapeseed in Various Countries

**Figure 3:**
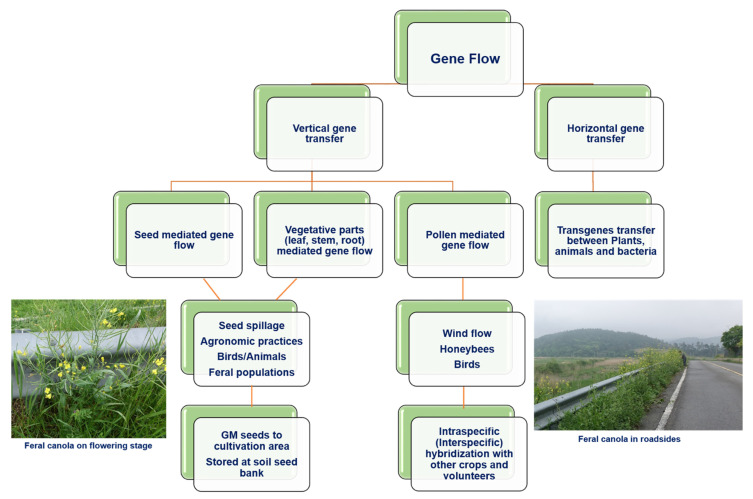
Overview of various methods of gene flow from GM crops.

As discussed above, rapeseed can produce wild populations in succeeding crops or appear as a volunteer outside of the crop area [31,42]. It has a number of wild relatives and is commonly found in Central Europe, which increases the chances of crossbreeding [30,33]. It can grow on both wasteland and cultivated land, forming persistent wild populations that can act as pollen donors and acceptors [31,32]. Most rapeseed plants beside the road have a high risk of spillage when seed sowing or harvesting equipment is transported, or when seeds are transported from fields or ports of import to processing facilities. The regional processes underlying the population dynamics of rapeseed have been extensively studied (Figure 3), including population statistics [43,44], seed sowing and harvesting machinery [44], and vehicle traffic [16,45]. According to the reports, gene flow through seeds can have a considerably larger impact on agriculture in terms of time and scale than gene flow through pollen [8,13] (Figure 3). Here, we further elaborate on the different types of unintentional environmental releases of GM rapeseed in countries where it is grown or imported (Table 2 and Table 3), as well as on the research trends in environmental risk assessment owing to unintentional environmental releases in major countries.

### 3.1. Japan

Rapeseed has been farmed in Japan for more than a century, resulting in huge wild populations. Japan’s annual consumption averages around 2.4 million tonnes [88]. The production and cultivation area in Japan increased dramatically after government-led public relations efforts in the 1930s [89]. Due to the rapeseed import permits, this expansion has slowed down since the late 1960s, and now Japanese consumption is mainly dependent on imports [49]. Japan currently produces 3580 tonnes per year, accounting for only 0.1% of total consumption [90]. Production is concentrated in only a few areas (1830 ha), such as Hokkaido, Japan’s northernmost island, where large-scale agricultural production systems are used [90]. Although production in Japan has nearly ceased, wild rapeseed populations can still be found in a variety of locations [46]. The majority of these are found along highways and rivers.

In Japan, the GM rapeseed volunteer was first reported in the year 2005 [46]. Herbicide (glufosinate and glyphosate)-resistant rapeseed was found in five major ports and roadsides of the Kanto region. According to trade statistics from Japan’s Ministry of Finance, 73% of the rapeseed imported into the country in 2004 came from Canada, 27% from Australia, and less than 0.002% from the US and Poland, respectively. Among them, 77% of Canadian rapeseed was GM rapeseed. Of all the rapeseed grown in Canada in 2001, 47% was glyphosate-resistant, 13% glufosinate-resistant, and less than 1% was bromoxynil-resistant [91]. The two prior herbicide-tolerant cultivars are grown in both Canada and the United States, but only a tiny amount of each has been imported to Japan. Since GM rapeseed is not commercially cultivated in Japan, its existence in major ports and along roadways is most likely the consequence of transportation spillage [46]. Some of the progeny of the transgenic rapeseed observed as a result of monitoring transgenic rapeseed in western Japan in 2005 possessed both glyphosate- and glufosinate-resistant transgenes [47]. It is possible that the two types of GM rapeseed plants crossed each other, since no double herbicide-tolerant transgenic strains of rapeseed have been developed for commercial purposes. This is thought to be the first time two herbicide-tolerant genes have been integrated through in-breeding. Imported rapeseed seeds are discharged from a number of major ports (Kashima, Chiba, Yokohama, Shimizu, Nagoya, Yokkaichi, Sakaisenboku, Kobe, Uno, Mizushima, Kitakyushu, Hakata) and transported to inland processing plants through several main national highways. Route 51 is one of the key transportation routes for rapeseed from Kashima Port to the Keiyo District in central Japan. In a recent investigation by Saji et al. [46], the existence of herbicide-resistant rapeseed plants was confirmed at various spots along this road.

Rapeseed plants were discovered annually, but the number of plants varied during the three-year study period. There were only 2162 in 2005, 4066 in 2006, and only 278 in 2007. It was assumed that the low number in 2007 was due to road construction. Individuals resistant to herbicides were found for three years in a row (26, 8, and 5 glyphosate-resistant individuals), but glufosinate-resistant plants were only discovered in 2005 (9 individuals). These plants are likely to have their origins from seeds spilled during cargo shipments at ports, as there is no potential natural seed supply of rapeseed near Route 51. Aono et al. [47] monitored the roadside of National Route 23 leading to Yokkaichi Port, in the area around this port, and along Route 23 for comparison with National Route 51 data, and discovered a substantial number of GM rapeseed fields with single herbicide tolerance qualities. Later, Aona et al. [51] found GM rapeseed with resistance to both glyphosate and glufosinate for consecutive years from 2005 to 2008. During the three-year monitoring period, no plants resistant to either herbicide were detected along Route 51 [49]. On the other hand, a small number of individuals with both herbicide tolerance features were discovered in some areas of National Road 23.

In order to elucidate the origin of wild rapeseed in Japan, Chen et al. [92] analyzed the wild rapeseed populations collected from various regions of Japan using several reliable and polymorphic-rich SSR markers. The results were compared with SSR marker-based genotyping data from NARO Genebank populations to investigate possible sources of wild rapeseed, also with the goal of determining the origin of GM wild rapeseed. The genotyping of 537 individuals (130 of which were determined to be GM) from different regions of Japan with 30 SSR markers revealed that 334 alleles were amplified and showed a moderate genetic diversity and high levels of inbreeding within the wild population. A population analysis using PCA analysis and the STRUCTURE program showed that 537 individuals could be assigned to eight genetic clusters, with very high genetic differences amongst individuals within the same geographic group. Many are closely related to the NARO gene bank’s rapeseed accessions, but some have unknown origins. The results for GM crops also show that they come from two separate places and have a significant degree of diversity, which can be explained by crossbreeding with neighbors and hybrid segregation. The findings of this study may aid in the better management of wild and GM rapeseed in Japan.

### 3.2. USA

GM rapeseed was initially approved for commercial release in the United States in 1998, and the majority (90%) of the area currently planted in the US has been genetically engineered for herbicide resistance [57,93]. Schafer et al. [57] investigated the extent of wild rapeseed populations in North Dakota, a significant rapeseed producing state in the United States. A roadside survey was undertaken, and commercially available test strips were employed to assess the distribution of transgenic rapeseed growing off-field in the United States. As a result, GM rapeseed escaped from North Dakota in large quantities. Rapeseed was found in 45% (288/34) of road surveys, of which 80% (231/288) carried at least one transgene, 41% (117/288) CP4-EPSPS alone, 39% (112/288) showed PAT only, and 0.7% (2/288) showed both types of herbicide resistance. Countries that cultivate GM rapeseed over a vast region, like the US, are notorious for scattering massive numbers of seeds before and after harvest [94]. When scattered seeds are buried, the seeds can enter into secondary dormancy [26,95]. Even if all native rapeseed is controlled before producing seeds in the first year of the following year, seedlings will continue to emerge from dormant seeds for several years, and they will become weedy plants [12,17,43,96]. In fact, four years following the 2007 harvest, wild rapeseed plants in California produced thousands of plants per hectare, despite the fact that no extra rapeseed seed was produced after the 2007 harvest [58].

### 3.3. Canada

GM rapeseed with glyphosate- and glufosinate-tolerant genes was first introduced in Canada. Immediately after the commercial launch, transgene escape and the occurrence of GM rapeseed on the roadways were reported [54,97]. Rapeseed was farmed on 5.5 million hectares in western Canada in 2005, with Saskatchewan accounting for half of that [30,98]. The presence and persistence of GM rapeseed wild populations in Canada is linked to truck transportation routes, such as those between fields and granaries. Yoshimura et al. [30] singled out the two main rural areas of Saskatchewan, where half of Canada’s rapeseed is grown (along railways and roads), and the west coast, the destination for most rapeseed and where the rapeseed is transported by rail. Rapeseed from the British Columbia port of Vancouver was investigated. As a result, transformants were found in two-thirds of the plants examined in Saskatchewan and Vancouver. A single transgenic *B. rapa* X *B. napus* hybrid was discovered beside the road in Vancouver, indicating that these two Brassica species are likely to hybridize. In 2006, over 80% of the rapeseed land had been converted and had developed resistance to the non-selective herbicides glyphosate (50%) and glufosinate (32%) [98]. Following the commercial approval of GM rapeseed, wild rapeseed plants are more prevalent on arable land, and the presence of numerous GM herbicide-tolerant (GMHT) traits may lead to transgene spread [33,98]. Many farmers are concerned with the inhibition of GMHT traits due to management issues with GMHT volunteers and the widespread cultivation of GM crops in western Canada [99]. Indeed, the prevalence of undesired GMHT traits in Canadian production systems and agricultural supply chains is inevitable [100]. Considering these concerns, a better understanding of the processes by which rapeseed plants and consequent GMHT transgenes move out of cultivation and spread to adjacent non-wild habitats is needed. Early studies on transgene escape from GM rapeseed focused on small experimental groups. Despite its ease of establishment in disturbed habitats, this population was unable to compete with encroaching perennial vegetation and quickly became extinct [101]. There is a growing recognition that landscape sizing is important to accurately characterize the extent of transgene escape and spread in areas where GM crops are cultivated. Knispel and McLachlan [17] investigated GM rapeseed that escaped along roadside and field edges from 2005 to 2007 in 12 locations in three agricultural landscapes in southern Manitoba, where GMHT resistant rapeseed is widely grown. The data were analyzed in order to investigate temporal changes at broad spatial scales and to identify factors influencing the distribution of escaped GM rapeseed from roadside and field-edge habitats within agricultural landscapes. To assess the possibility of seed dispersal among escaped populations, we evaluated the relative spatial distributions of roadside and field-edge rapeseed. As a result, the density of escaping rapeseed varied over time and space in both roadside and field-edge habitats, despite the increased number of GMHT plants (93–100%). The escaped rapeseed was positively affected by the presence of cropland and adjacent fields planted with rapeseed. Its escapes within roadside habitats were also strongly related to large-scale variables, such as road surface (indicative of traffic intensity) and distance to the nearest grain elevator [17,56]. Conversely, within field edges, rapeseed density was affected by local crop management practices such as lawn mowing, soil disturbance, and herbicide application. Escaped rapeseed populations persisted on large spatial and temporal scales, and the low density of a given landscape or year did not indicate an overall extinction. Escaping rapeseed from field-edge habitats generally results from local sowing and management activities occurring at a field scale, but the distribution patterns within roadside habitats are largely determined by seed transport occurring at the landscape scale and larger local scales. This widespread dispersal has the potential to undermine field-scale management practices to eliminate the escape and field GM rapeseed populations.

### 3.4. Switzerland

GM plants have never been produced in Switzerland and are prohibited until the end of 2017 due to a legal moratorium by FOAG. Unlike in the EU, Swiss federal legislation considers unintentional releases (i.e., spills and dispersions) of GM plants to be harmful to the environment. Since 2008, no GM crops have been imported into Switzerland. Imports of GM rapeseed for human consumption are also banned. Switzerland is therefore considered to be free from growing and importing GM crops. However, it produced approximately 70,000 tonnes of rapeseed in 2009. About 11,000 tonnes were imported, mainly for the production of cooking oil and biofuels. Imports came mainly from Hungary, Romania, Austria, Germany, and the Balkans. Switzerland did not import rapeseed from large-scale GM rapeseed producing countries such as Canada or the US, but it imports a large quantity of wheat from Canada. Bans on production and importation, however, were ineffective in preventing the spread of GM rapeseed. 

GM rapeseed was found most frequently at ship loading docks, indicating that ship freight transport is the main entry route for GM rapeseed. Railroad lines are densely connected habitats, with a significant risk of unintentional release of rapeseed due to seed runoff during transportation. Glyphosate is frequently used to control the vegetation beside railroad tracks, increasing the possibility of resistant plants. Schoenenberger and D’Andrea [59] studied the presence of glyphosate-resistant GM rapeseed in 77 railway regions in the Principality of Liechtenstein, centered on a Swiss railway station. Since Switzerland does not import or cultivate GM rapeseed, the aim of the investigation was to detect the unintentional release of transgenic plants. As a result, a total of 50 plants expressing the CP4-EPSPS protein were detected in four regions, one in Lugano and three in Basel. Schulze et al. [60] investigated the distribution of wild and GM rapeseed and the possibility of transgene flow from GM rapeseed to wild non-GM rapeseed and related plant species in Basel’s Rhine port. As a result, the presence of GT73 (GM rapeseed) was confirmed at all previously documented sample locations within the Rhine port, as well as at new sampling locations. At five sampling locations in the Rhine port, they discovered glufosinate-resistant GM events MS8xRF3, MS8, and RF3 (all traded as InVigor, Bayer). This is the first time in Europe that wild MS8xRF3, MS8, or RF3 plants have been discovered. A PCR analysis of the seeds showed that GT73 crossed into two non-GM rapeseeds, but no crosses of the transgene into related wild species were observed. It also identified no hybrids between rapeseed and related species. This suggested that the wheat imports from Canada were a possible source of GM rapeseed (GT73, MS8, RF3, MS8, and RF3) contamination in the Rhine port of Basel and in the processing sites of two grain mills in Switzerland [60]. Later, a study by Hecht et al. [61] compared surveys along rail routes from the Swiss borders with Italy and France to respective rapeseed processing plants in southern and northern Switzerland (Ticino and Basel regions) with random sampling sites. More numbers were found at the same risk hotspots, and the GM rapeseed strain GT73 carrying the glyphosate-resistant transgene, gox, and CP4-EPSPS was detected at three locations in both monitored regions (Ticino, 22 plants; Basel region, 159). 

Railroads are an ideal system in which herbicide-tolerant GM plants are established and spread as a result of high selective pressures favoring herbicide resistance, resulting in increased difficulties in keeping infrastructure free of weeds. When sexually compatible species, that is, closely related species of the same species or genus, grow in the same place, a crop-to-wild gene flow can occur. Moreover, the capillary presence of railways in agricultural landscapes poses a potential source of pollution for GM-free agriculture. Wild GM rapeseed plants were discovered growing on railway lines and in port areas in four Swiss regions in 2011 and 2012 [60,61]. The glyphosate-resistant GM event GT73 was detected in all GM rapeseed (Roundup Ready, Monsanto). The Rhine Port and the St. John Freight Train Station in Basel were the most affected sites. Vegetation growth at both sites is controlled by regular glyphosate treatments. The selective pressure by glyphosate promotes the growth of GT73 rapeseed and increases the risk of escape of glyphosate-resistant transgenes through hybridization and the invasion into related species. When established contamination levels are biologically insufficient to prevent future environmental contamination, our findings should enable the development of carefully coordinated monitoring designs to detect events that can lead to rapid settlement and population growth.

### 3.5. Australia

Australia has been hesitant about the introduction of GM rapeseed. The Australian Office of the Gene Technology Regulator (OGTR) granted commercial release permits for Roundup Ready^®^ and InVigor^®^ rapeseed cultivars in 2003, although they were not cultivated commercially until 2008 [83]. Between 2003 and 2007, the Australian rapeseed sector worked hard to design and implement procedures and methods that would efficiently separate non-GM from GM rapeseed. In 2009, the Roundup Ready varieties of GM rapeseed were cultivated on 9600 hectares that produced 9336 tonnes of grain, whereas in 2010, a total of 317 growers chose to plant about 72,000 hectares with a production of 49,000 tonnes. GM rapeseed spills have been confirmed in Western Australia [83]. However, the persistence of GM rapeseed outside of agricultural fields in disturbed regions such as roadsides or natural habitats has not been explored. Simultaneously, Busy and Powles, [84] performed a field study for four consecutive years (2009–2013) to identify the persistence of GM rapeseed in natural areas and roadsides of Western Australia. A roadside study conducted in October 2012 at a major Perth Metro grain storage facility found that volunteer rapeseed plants were growing on a 3500 m roadway transect that connected to this grain-receiving site [84]. The initial propagule of a transgenic GR rapeseed population in a natural environment decreased over time and might persist for up to three years after being unintentionally released outside the agricultural areas.

### 3.6. Argentina

Argentina’s rapeseed production is limited, with a planted area of about 36,000 ha and a yield of 59,000 tonnes in the past decade. It mainly depends on the import from the marketing countries like USA, Canada, Australia, Germany, and Sweden [102]. GM rapeseed has never been cultivated in Argentina, and it has been prohibited since 1997, when the national Secretariat of Agriculture banned the experimental production of GT73 GM rapeseed. In 2007, the prohibition was expanded, making it illegal to import GM rapeseed for cultivation or marketing, and any import of rapeseed had to be accompanied by a GMO-free analysis from the exporting nation. Moreover, there is no field trail for the GM rapeseed in the country. However, in 2012, few agricultural fields with no recent records of cultivation of rapeseed were invaded with transgenic rapeseed plants with glyphosate applications in the southeast of Buenos Aires province [62]. In the fields of soybean and other crops, the transgenic rapeseed was found as weeds. The immunological and molecular analyses found that the accessions were from the GT73 transgenic event. Since then, the production and import of GM rapeseed is forbidden in Argentina, and the cause of this incident is unknown. This finding indicates that glyphosate resistance originates in the nation through the illegal cultivation of transgenic rapeseed or as seed contaminants in imported rapeseed cultivars or other seed imports [62]. The presence of these populations also raises concerns about the possibility of hybridization with wild-related species, particularly *B. rapa*, because this species is common in the area where GM herbicide-resistant *B. napus* was discovered, which is the main reason cited by the national authorities to prohibit the growing and import of GM rapeseed varieties.

### 3.7. European Union

Despite the fact that GM rapeseed was never approved for cultivation in the European Union, 11 countries have conducted field testing, and a few of them found the spread of GM rapeseed (Table 3). Transport and handling have been identified as key factors in the spread of rapeseed [16,77]. European Union countries such as France [16,43,44,45], Germany [18,72,74,76,103], the Netherlands [85], the United Kingdom [12,77], and Austria [66,67,68] were reported to have persistently detected rapeseed for many years outside of the plantations, along transport routes such as railways and roads. However, there is no systematic screening of GM admixtures in imported seeds, their losses, or establishment in the EU, even though the screening for GM feral rapeseed was done regularly to assess the unintentional release of the rapeseed in the respective countries.

#### 3.7.1. Austria

To meet market demand, Austria relies on rapeseed imports. The majority of imported rapeseed seeds come from Europe (namely Hungary, Serbia, and Slovakia), and a small amount from Chile and New Zealand. Austria is well connected with eight neighboring countries through railway networks, and rapeseed seeds are delivered there every year. Since Austria is located in the center of Europe and serves as a node point for transportation and international goods transfer, Pascher et al. [66,67,68] chose it as a study site for rapeseed spills. In the study, they assessed the mid- to long-term spillover potential of rapeseed seeds using field data collected along transport routes and from loading and handling sites in Austria, where the import of GM rapeseed is prohibited. As a result, feral rapeseed was found in 2014 and/or 2015 in 44 of the 60 sites surveyed [68]. This included several locations outside of rapeseed production sites, where feral rapeseed is likely to have originated from imported rapeseed seed spillage rather than the transfer of Austrian rapeseed seeds [68]. The majority of the populations were present in both years, suggesting that they have persisted over the years. The plants were flowering, had already developed viable seeds, and showed a high vigor, and the number of plants was consistently higher than in regions without growing areas. In the 60 observation sites, two species were detected in 25 sample sites, with up to five possible hybridization partners discovered in one sample site. The relatives most frequently observed were *Sinapis arvensis* (21 sites) and *Diplotaxis tenuifolia* (20 sites), but no hybridization events were witnessed [104]. Previously, Moser et al. [104] used a high-resolution spatially explicit simulation of 140 distinct coexistence scenarios within six primary rapeseed cropping districts of Austria (2400 km^2^) to investigate the effects of GM rapeseed cultivation for biodiesel production. As a result, they concluded that GM rapeseed application for biofuel feedstock production is not a viable option under the present regulatory requirements and crop production conditions, neither for Austria nor for nations with similar land ownership and land use patterns.

#### 3.7.2. France

Surveys of rapeseed populations in many nations have revealed that seed spillage from grain trucks plays a significant role in the spread of rapeseed, which is strongly linked to the transportation network. In France, seeds transported by trailers and vehicles were determined to be the source of 15% of the feral populations [14]. A study aimed at identifying feral rapeseed in the production area region centered around the village of Selommes (Loir-et-Cher, central France) and on a silo, to which the majority of the farmers who own fields in the area deliver their products [16,43,44,69,70,104]. The former survey of feral rapeseed populations had indicated that they can be persist for up to 8 years in semi-natural habitats [43]. The use of a stage-structured integro-differential model for the prediction of feral rapeseed on the road verge indicated the invasiveness of GM rapeseed feral populations. It was primarily determined by a few key life-cycle transitions as well as the presence of long-distance seed dispersions [45,105]. Many feral rapeseed populations (about 35–40%) arose from seed dispersal from adjacent fields. Seed dispersion occurred during harvesting, rather than during the sowing period. Almost 15% of these populations were ascribed to dispersion through seed transportation by trailers and trucks [16,44]. The other half of the populations was followed, mostly through persistent seed banks. Despite the fact that there were no records of seed banks in the road verges, this was more surprising. A study with machine-learning approaches for the prediction of the origin of feral rapeseed populations was performed for the large data set collected in a 4-year survey [44]. Concurring with previous studies, the results showed that the seed dispersion is mostly caused by the seed transport and persistence. It is important to measure the number of seeds dispersed through spillage during transport in order to get effective management processes [70]. Establishing seed trap-sites around the road verges resulted in the prediction of the possible total spillage around the road verges. The study concluded that the amount of seed spilled is positively correlated with the area of rapeseed cultivation [70]. Further, studying the genetic diversity among the plants growing in a field for a long time will potentially reveal the origin of feral rapeseed populations and be useful for the containment of feral GM rapeseed plants [19,71]. The genetic studies revealed the diversity that exists among the different feral populations in the area with field-grown plants. These studies should be incorporated with field modeling studies for the effective containment of GM rapeseed on cultivation land and road verges.

#### 3.7.3. Germany

Although the EU has maintained a ban on the cultivation of GM rapeseed, the import and processing of whole and viable transgenic seeds for various herbicide-tolerant lines has been permitted [106]. Therefore, the possibility of transgene escape is quite high, and it was regularly assessed for their unintentional release to the environment. The field survey during the years 2004–2007 investigated the origin, persistence, and genetic diversity of feral rapeseed populations in a 30-km radius surrounding the city of Osnabrück (Lower Saxony) and covered both urban and rural regions of northwest Germany [76]. This study reported that the majority of the locations studied (72%) had been occupied by rapeseed for at least two years. The proportion of feral populations that produced seeds varied from year to year (30–48%) and was greater than in previous research. The genetic diversity of wild rapeseed populations was greater than that of the examined commercial cultivars [76]. Transgene escape might be facilitated by feral populations of agricultural plants. Self-sustaining feral populations can improve the transgenic persistence outside of cultivation by increasing the intraspecific and interspecific gene flow [76]. If GM rapeseed cultivars were introduced into agricultural operations, it would rapidly result in the creation of mixed GM and non-GM feral populations in northern Germany. Wurbs et al. [107] performed a database and literature survey to detect the hybridization potential of GM rapeseed in the state of Brandenburg. They discovered that Brandenburg has a high risk of transgene escape and proposed that regular monitoring of GM rapeseed be done using a targeting approach. Model-based assessments were successfully extrapolated to the regional level using a set of ecological indicators that allowed them to analyze the possible consequences of GM rapeseed introduction in the fields [73,108,109]. The extrapolation technique provided various combined characteristics to analyze GMO impacts on broad geographical scales in terms of persistence and dispersion. Franzaring et al. [18] conducted a field survey in the vicinity of big oil mills and seed processing industries at the harbors along the river Rhine. Individuals or large groups of feral rapeseed plants were found in all the nine locations studied, but only one GM rapeseed (GT73) plant out of 1918 tested was confirmed. The findings indicated that herbicide-tolerant GM rapeseed had not spread to that point [18]. They also concluded that a periodic monitoring of feral rapeseed is important to ensure the absence of GM feral rapeseed and the potential harmful impacts of GM plants in the future. Over 300 field experiments with 15 distinct GM rapeseed events (GS 40/90 pHoe/Ac, Liberator C/6Ac, and MS8/RF3) were conducted at 88 different locations in Germany between the years 1994 and 2007. Among them, glufosinate-resistant rapeseed was planted in 247 fields at 62 distinct locations across the country. For the first time, the monitoring data demonstrated the survival of GM rapeseed in arable fields 13 and 15 years following (one-time) agronomic testing [21]. Despite the persistence, the study showed no spatial dispersion of herbicide-resistant GM rapeseed in the environment of the release sites over many years.

#### 3.7.4. Sweden

Potential GM volunteers were discovered in Sweden after 10 years of a field trial of GM rapeseed [110]. Previously, in 1995, three GM rapeseed lines were field-trialed at Lonnstorp Experimental Farm, Sweden. The trail was harvested in autumn 1995. Later, there was no cultivation of rapeseed on the farm, but wheat, barley, and sugar beet were cultivated in the years 1996–2005. The fields were perfectly managed during these years, with proper agronomical practices. Despite the practices and control measures, some volunteers were still observed after 10 years. Among the identified volunteers, 15 plants survived in the herbicide applications, which shows they were still holding the transgene [110]. 

## 4. Management Practices to Avoid the Unintentional Release of GM Rapeseed

As elaborated above, the number of cases and the distribution of both GM and non-GM feral rapeseed are very common. However, there is no report on their negative effects on the environment, only on agriculture. Since rapeseed becomes weedy, growing on roadsides and other agricultural fields, it is very important to manage its spread. The containment of the spread of rapeseed seeds will always be challenging. However, seed spillage could be reduced if grain trucks were covered and filled with less seeds. Further, seed loss might be reduced by shortening the distance between the fields and the processing places [68]. During the transport of grains with trucks, there is a higher possibility of spilling rapeseed due to the wind flow, so covering the grains and filling the truck with less seeds could effectively reduce the seed spillage. Moreover, while reducing the distance between the fields and the processing places, we can minimize the area of spillage, thus achieving an effective management of the feral rapeseeds. Devos et al. [111] identified important management practices, including:

1. Controlling the seed production of *Brassica* crops in isolated areas in order to meet conventional purity standards for certified seed,
2. Cultivating certified seed to reduce the risk of off-types with altered traits,
3. Isolating fields of GM rapeseed cultivars to limit out-crossing, and
4. Harvesting GM rapeseed at the right development stage of the crop with well-adjusted settings.
5. To ensure the maximum germination of spilled seeds, avoid deep soil inversion for at least 3–4 weeks after harvest and use ploughing as the primary tillage method before planting the following crop.
6. Applying suitable herbicide applications and planting a competitive crop following rapeseed to ensure an effective weed control in subsequent harvests,
7. Rotating rapeseed in a lengthy and diversified cropping sequence to decrease the seed bank over time, and
8. Preserving precise on-farm records to track a plot’s history.

It will be critical to ensure that the suggested on-farm and off-farm measures are widely fulfilled [31,111]. Herbicide use, cultivation, and rotational practices are still effective management tools for farmers, even for herbicide-tolerant rapeseed. It is likely that these herbicides, applied to transport routes and other managed areas, will do more environmental harm than the GM rapeseed. Hence, it is important to minimize the use of herbicides in the environment. Garnier and Locomte [105] found that the possibility of wild GM rapeseed colonizing roadside verges under selection pressure is real. Long-distance dispersion events should thus be included in models of gene dispersal in rapeseed to give useful information for determining optimal management methods. Pascher et al. [68] recommended measures to minimize and mitigate the spillage of imported crops, such as rapeseed, including seed packaging methods during transport, such as the use of sealed bags, enhanced testing of grain cargo, the management of runoff weeds around transport routes, the implementation of a monitoring program for imported herbicide-resistant crops, and the need for extensive cooperation in both research and practice for interdisciplinary exchange and the efficient management of wild crops along transport routes. Moreover, they emphasized the need for harmonization to establish successful access standards and international guidelines for the transport of crops (especially needed with rapeseed seeds). 

## 5. Conclusions and Future Perspectives

In conclusion, the unintentional release of GM rapeseed mainly occurs due to the seed spillage during harvesting and storing in the soil seed banks, and the seed spillage during the importation of the GM rapeseed and their transportation along the road verges. After a while, they become feral populations. Companies, government organizations, and port and road authorities should strengthen environmental monitoring and management, as well as put in place measures to prevent the spill, spread, and persistence of GM rapeseed near port regions. A number of studies have concluded that a mechanical or chemical control of roadside feral plants can be attained at a local scale [31,33,48], provided that monitoring systems are in place to detect where significant populations of feral rapeseed are present [32,112,113], and that can be an effective management strategy. In addition, as a successful coexistence measure, policies should be put in place to prevent the contamination of GM crops. The approach may be a multi-step approach that can be executed at farm- and landscape-level organizations, such as seed developers and GM and non-GM farmers. It proposes that numerous stakeholders, such as processors, transporters, and suppliers, be consulted, and that adopting GM crops necessitates a thorough examination of the risks and obligations associated with this new technology.

## Figures and Tables

**Table 1:** Import of rapeseed in major importing countries.

S. No	Countries	Importing Quantity (10^4^ Tonnes)
1.	Germany	574.637
2.	China	475.6582
3.	Belgium	258.8239
4.	Japan	233.74
5.	Mexico	143.6321
6.	France	94.0338
7.	Pakistan	80.8421
8.	United Arab Emirates	73.6002
9.	Poland	71.7704
10.	Netherlands	68.7646
11.	United States of America	62.917
12.	Czechia	28.8407
13.	Austria	28.6828
14.	Belarus	26.1836
15.	United Kingdom	19.7132
16.	Denmark	16.614
17.	Portugal	15.8598
18.	Canada	15.5105
19.	Sweden	12.4454
20.	Nepal	9.0375
21.	Bangladesh	8.9847
22.	Hungary	7.7505
23.	Republic of Korea	0.5601
24.	Switzerland	0.4906
25.	Australia	0.1176

Source: FDA Statistics (https://www.fao.org/faostat/en/#data; accessed on 18 November 2021).

**Table 2:** Case studies on the unintentional release of GM rapeseed in the environment.

Nation	Year of Study	Region	Escaped Transgene	Hybridization	Comments	References
Japan	2004	Kashima, Kobe, Kanto R51 Kanto R124	PAT, EPSPS	N/A	First published example of feral, transgenic populations occurring in a nation where the transgenic crop has not been cultivated commercially	[46]
		Chiba, Nagoya Yokkaichi	EPSPS			
	2005	Kashima, Chiba, Yokohama, Shimizu, Nagoya, Yokkaichi, Sakai-senboku, Kobe, Uno, Mizushima, Kita-Kyushu, Hakata	PAT, EPSPS	Inter-Specific Hybridization with *B. rapa*, *B. juncea*	First report identifying the outcrossing between different Brassica species.	[47]
	2004–2007	Fukushima, Mizushima	EPSPS	N/A	Seed spillage during transportation is the main cause for the gene transfer	[48]
		Kashima, Chiba, Nagoya, Yokkaichi, Hakata	PAT, EPSPS			
		Yokohama, Shimizu, Ooita, Nagasaki	PAT			
	2005–2007	Kanto Route 51	EPSPS (2005~2007) PAT(2005)	N/A	Detailed report on seed spillage during transportation as the main cause for the gene transfer	[49]
	2004–2005	19 sites around Kashima sea port	PAT, EPSPS	N/A	Found GM rapeseed in only 2 sites	[50]
	2005–2008	Kashima, Chiba, Yokohama, Shimizu, Nagoya, Yokkaichi, Sakai-senboku, Kobe, Uno, Mizushima, Kita-Kyushu, Hakata	EPSPS	Inter-Specific Hybridization with *B. rapa*	Origin of double resistance unclear	[51]
	2006–2011	Kashima, Chiba, Yokohama, Shimizu, Nagoya, Yokkaichi, Sakai-senboku, Kobe, Uno, Mizushima, Tobato, Hakata	EPSPS	N/A	Chiba, Yokkaichi, and Hakata were the hotspots for the feral rapeseed populations	[40]
	2005–2014	Kanto Route 51	EPSPS, PAT	N/A	Ten years of seed spillage during transportation is the main cause for the gene transfer	[52]
Canada	1996–1998	Alberta	EPSPS	N/A	Neighboring field, multiple herbicide resistance	[53]
	2002	Saskatchewan	PAT, EPSPS	N/A	Neighboring field, multiple herbicide resistance, double resistance in seed lots	[33]
	2002	Western Canada	PAT, EPSPS	N/A	Double-resistant seed lots	[54]
	2000	Québec	EPSPS	Inter-Specific Hybridization with *B. rapa*	Commercial fields, no escape to *Raphanus raphanistrum*, *Sinapis arvensis*, or *Erucastrum gallicum*	[22]
	2005	Vancouver	EPSPS	Inter-Specific Hybridization with *B. rapa*	High probability of hybridization between these two Brassica species	[30]
	2003	Québec	PAT, EPSPS	Inter-Specific Hybridization with *B. rapa*	Double resistance by transgene flow in escaped populations	[20]
	2005	Québec	EPSPS	Inter-Specific Hybridization with *B. rapa*	Persistence over 6 years	[55]
	2004–2006	Manitoba	PAT, EPSPS	N/A	Double resistance by transgene flow in escaped populations	[56]
	2005–2007	Manitoba	PAT, EPSPS	N/A	Agricultural transport and landscape-scale cropping pattern are the key determinants.	[17]
USA	2008–2009	North Dakota	PAT, EPSPS	N/A	Double resistance in feral rapeseed at the roadways	[57]
	2007–2011	Butte county farm (California)	EPSPS	N/A	Glyphosate-resistant rapeseed in the fields	[58]
Switzerland	2011	Swiss railway station, Basel, Liechtenstein	EPSPS	N/A	Four GM rapeseed were identified in 2 sites	[59]
	2012	Basel’s Rhine port	PAT, EPSPS	N/A	Discovered glufosinate-resistant GM events MS8xRF3, MS8, and RF3	[60]
	2010–2012	Rail roads along the country (Basel)	PAT, EPSPS	N/A	Strain GT73 carrying the glyphosate resistance transgene, gox, and CP4-EPSPS were detected	[61]
Argentina	2012	Southeast of Buenos Aires province	EPSPS	N/A	Transgenic rapeseed (GT73) was identified	[62]

**Table 3:** Case studies on feral rapeseed populations in the environment.

Nation	Year of Study	Region	Comments	References
Belgium	2007–2008	Roadsides in Wallonia	-	[63,64]
	2009	Port areas of Antwerpen, Gent, Izegem, and Kluisbergen	-	[65]
Austria	1998–1999	Burgenland, Waldviertel, and Innviertel	Field evaluation and genetic variation analysis among the feral populations	[66,67]
	2015–2016	60 sites all over Austria considers transportation routes (railways, roads) and loading sites such as railway stations, switch yards, ports, oil mills, and processing companies.	Feral rapeseed found in 44 of the 60 sites surveyed	[68]
Denmark	2005–2006	Mid-Jutland	Population dynamics of feral rapeseed	[12]
France	1996–1997	Roadways in Selommes	Origin and persistence of feral rapeseed populations	[43]
	2000–2003	Village in Selommes	Population dynamics of feral rapeseed and modeling studies	[16,44]
	2000–2005	Roadways and field edges in Selommes	Population dynamics of feral rapeseed	[12,69]
	2002–2006	Village in Selommes	Population dynamics of feral rapeseed	[70]
	2010	Village in Selommes	Genetic variation analysis among the feral populations	[71]
Germany	2001–2003 2005	Bremen	Population dynamics of feral rapeseed	[12,72,73]
	2002–2005	Braunschweig	Population dynamics of feral rapeseed	[12,74,75]
	2004–2007	Lower Saxony	Population dynamics of feral rapeseed	[76]
	1998–2015	Saxony-Anhalt	Dynamics of feral GM rapeseed events (MS8/RF3, GT73, GS 40/90, and MS1/RF1) in different time periods and long-term persistence	[21]
United Kingdom	1993–2002	Roadways in southern England	Dynamics of feral rapeseed in roadways	[77,78]
	1993–1994 2004	Tayside region (Scotland)	Field survey in roadways and genetic variation analysis	[12,79,80,81]
	1994–2000	Fields across the England	Distribution and dynamics of feral rapeseed	[82]
Australia	2009–2011	Fields in western Australia and Albany Highway	Step wise adoption of GM rapeseed in agricultural fields and their persistence	[83]
	2009–2013	Roadsides of western Australia	Occurrence of feral rapeseed in roadsides and grain-receiving sites	[84]
The Netherlands	2008–2009	Ports of Rotterdam and Amsterdam	Distribution and dynamics of feral rapeseed	[85]
New Zealand	2003, 2005	Canterbury (South Island)	Distribution of feral rapeseed in road verges, drainage ditches, channels, natural watercourses, shelterbelts, and wasteland	[86,87]

## Data Availability

Not applicable.

## Supplementary Materials

The following are available online at https://www.mdpi.com/article/10.3390/biology10121264/s1, Table S1. Details of various GM rapeseed events and their properties.
