# Supplementary material for: A Review of the Unintentional Release of Feral Genetically Modified Rapeseed into the Environment
Source: Biology (Basel). 2021 Dec 3;10(12):1264. doi: 10.3390/biology10121264 (PMC8698283; doi:10.3390/biology10121264)
Supplement: Supplementary file 1 [file biology-10-01264-s001.zip › biology-1426964-supplementary.pdf]

Table S1. Details of various GM rapeseed events, and their properties

Event	Developer	GM traits	Basic Information				Authotization			
			Gene Introduced	Gene Source	Product	Function	Country	Food	Feed	Cultivation
23-18-17	Monsanto (CGN-89111-8)	Modified oil/fatty acid, Antibiotic resistance	te<br>nptII	*Umbellularia californica* (bay leaf)<br>*Escherchia coli* Tn5 transposon	12:0 ACP thioesterase enzyme<br>Neomycin Phosphotransferase II enzyme	Increases the level of triacylglycerides containing esterified lauric acid(12:0)<br>Allows transformed plants to metabolize neomycin and kanamycin antibiotics during selection	Canada<br>United States	1996	1996	1996<br>1994
23-198	Monsanto (CGN-89465-2)	Modified oil/fatty acid, Antibiotic resistance	te<br>nptII	*Umbellularia californica* (bay leaf)<br>*Escherchia coli* Tn5 transposon	12:0 ACP thioesterase enzyme<br>Neomycin Phosphotransferase II enzyme	Increases the level of triacylglycerides containing esterified lauric acid(12:0)<br>Allows transformed plants to metabolize neomycin and kanamycin antibiotics during selection	Canada<br>United States	1996<br>1995	1996<br>1995	1996<br>1994
61061 (DP-ø61ø61-7)	DuPont	Glyphosate herbicide tolerance	Gat4621	*Bacillus licheniformis*	Glyphosate N-acetyltransferase enzyme	Catalyzes the inactivation of glyphosate, conferring tolerance to glyphosate herbicides	Canada	2012	2012	2012
73496 (DP-ø61ø61-7)	DuPont	Glyphosate herbicide resistance	Gat4621	*Bacillus licheniformis*	Glyphosate N-acetyltransferase enzyme	Catalyzes the inactivation of glyphosate, conferring tolerance to glyphosate herbicides	Australia<br>Canada<br>Japan<br>Malaysia<br>Mexico<br>New Zealand<br>Philippines<br>South Africa<br>South Korea<br>Taiwan<br>United States	2014<br>2012<br>2014<br>2018<br>2012<br>2014<br>2019<br>2016<br>2015<br>2016<br>2012	2016<br>2012<br>2015<br>2018<br><br><br>2019<br>2016<br>2015<br><br>2012	2016<br>2012<br>2015<br><br><br><br><br><br><br><br>2013
73496x RF3 (DP-ø73496-4 x ACS-BNøø3-6	DuPont	Glufosinate herbicide tolerance, Glyphosate Herbicide	gat4601<br>Bar	*Bacillus licheniformis*<br>*Streptomyces hygroscopicus*	Glyphosate N-acetyltransferase enzyme<br>Phosphinothricin N-acetyltransferase	catalyzes the inactivation of glyphosate, conferring tolerance to glyphosate herbicides<br>eliminates herbicidal activity of glufosinate (phosphinothricin)	Japan<br>Mexico<br>South Korea<br>Taiwan	2017<br>2018<br>2017<br>2018	2016<br><br>2017 	2017

		Tolerance, Fertility restoration	barstar	*Bacillus amyloliquefaciens*	(PAT) enzyme<br>Barnase ribonuclease inhibitor	herbicides by acetylation<br>restores fertility by repressing the inhibitory effect of barnase on tapetum cells of the anther				
DHA canola (NS-B5øø27-4)	Nuseed Pty Ltd	Glufosinate herbicide tolerance, Modified oil/fatty acid	Lackl-delta12D<br>Picpa-omega-3D<br>Micpu-delta-6D<br>Pyrco-delta-6E<br>Pavsa-delta-5D<br>Pavsa-delta-5E<br>Pavsa-delta-4D<br>pat	*Lachancea kluyveri*<br>*Pichia pastoris*<br>*Micromonas pusilla*<br>*Pyramimonas cordata*<br>*Pavlova salina*<br>*Pyramimonas cordata*<br>*Pavlova salina*<br>*Streptomyces viridochromogenes*	Delta-12-desaturase<br>Delta-15-/omega-3-desaturase<br>Delta-6-desaturase<br>Delta-6-elongase<br>Delta-5-desaturase<br>Delta-5-elongase<br>Delta-4-desaturase<br>Phosphinothricin N-acetyltransferase(PAT) enzyme	Converts oleic acid to linoleic acid<br>Converts linoleic acid to α-linolenic acid<br>Converts α-linolenic acid to stearidonic acid<br>Converts stearidonic acid to eicosatetraenoic acid<br>Converts eicosatetraenoic acid to eicosapentaenoic acid<br>Converts eicosapentaenoic acid to docosapentaenoic acid<br>Converts docosapentaenoic acid to docosahexaenoic acid<br>eliminates herbicidal activity of glufosinate(phosphinothricin)herbicides by acetylation	Australia<br>New Zealand<br>United States	2018<br>2018 	2018 	2018<br><br>2018
GT200 (RT200) (MON-89249-2)	Monsanto Company	Glyphosate herbicide tolerance	cp4 epsps (aroA:CP4)<br>goxv247	*Agrobacterium tumefaciens* strain CP4<br>*Ochrobactrum anthropi* strain LBAA	herbicide tolerant form of 5-enolpyruvulshikimate-3-phosphate synthase (EPSPS) enzyme<br>glyphosate oxidase	decreases binding affinity for glyphosate thereby conferring increased tolerance to glyphosate herbicide<br>confers tolerance to glyphosate herbicides by degrading glyphosate into aminomethylphosphonic acid (AMPA) and glyoxylate	Canada<br>Chile<br>Japan<br>United States	1997<br><br>2001<br>2002	1997<br><br>2003<br>2002	1997<br>2007 *<br>2006<br>2003
GT73 (RT73) (MON-øøø73-7)	Monsanto Company	Glyphosate herbicide tolerance	cp4 epsps (aroA:CP4)<br>goxv247	*Agrobacterium tumefaciens* strain CP4<br>*Ochrobactrum anthropi* strain LBAA	herbicide tolerant form of 5-enolpyruvulshikimate-3-phosphate synthase (EPSPS) enzyme<br>glyphosate oxidase	decreases binding affinity for glyphosate thereby conferring increased tolerance to glyphosate herbicide<br>confers tolerance to glyphosate herbicides by degrading glyphosate into aminomethylphosphonic acid (AMPA) and glyoxylate	Australia<br>Canada<br>China<br>European<br>Union<br>Japan<br>Mexico<br>New<br>Zealand<br>Philippines	2000<br>1994<br>2002 *<br>1997 *<br>2001<br>1996<br>2000<br>2003 *<br>2014<br>2003	 1995<br>2002 *<br>2007 *<br>2003<br><br><br>2003 *<br>2014<br>2005	2003<br>1995<br><br><br><br>2006

							Singapore<br>South Korea<br>Taiwan<br>United States	2015<br>1995		 <br><br>1999
HCN 10 (Topas 19/2)<br>(not available)	BASF	Glufosinate herbicide tolerance , Antibiotic resistance	bar<br><br><br>nptII *	Streptomyces hygroscopicus<br><br>Escherichia coli Tn5 transposon	phosphinothricin N-acetyltransferase (PAT) enzyme<br>neomycin phosphotransferase II enzyme	eliminates herbicidal activity of glufosinate (Phosphinothricin) herbicides by acetylation<br>Allows transformed plants to metabolize neomycin and kanamycin antibiotics during selection	China<br>Japan	2002 *<br>2001	2002 *<br>2003	 2007
HCN28 (T45)<br>(ACS-BNøø8-2)	Bayer CropScience	Glufosinate herbicide tolerance	pat (syn)	synthetic form of pat gene derived from Streptomyces viridochromogenes strain Tu494	phosphinothricin N-acetyltransferase (PAT) enzyme	eliminates herbicidal activity of glufosinate (Phosphinothricin) herbicides by acetylation	Australia<br>Canada<br>China<br>European Union<br>Japan<br>Mexico<br>New Zealand<br>South Korea<br>United States	2002<br>1997<br>2002 *<br>2009 *<br><br>2001<br>2001<br>2002<br><br>2005<br><br>1997	 1996<br>2002 *<br>2009 *<br><br>2003<br><br><br><br>2005<br><br>1997	2003<br>1996<br><br><br><br>2007<br><br><br><br><br><br>1998
HCN28 x MON88302 (ACS-BNøø8-2 x MON-883ø2-9)	Bayer CropScience	Glufosinate herbicide tolerance , Glyphosate herbicide tolerance	pat (syn)<br><br><br><br><br>cp4 epsps (aroA:CP4)	synthetic form of pat gene derived from Streptomyces viridochromogenes strain Tu494<br>Agrobacterium tumefaciens strain CP4	phosphinothricin N-acetyltransferase (PAT) enzyme<br><br>herbicide tolerant form of 5-enolpyruvulshikimate-3-phosphate synthase (EPSPS) enzyme	eliminates herbicidal activity of glufosinate (Phosphinothricin) herbicides by acetylation<br><br>decreases binding affinity for glyphosate thereby conferring increased tolerance to glyphosate herbicide	Australia	2016	2016	2016
HCN92 (Topas 19/2)<br>(ACS-BNøø7-1)	Bayer CropScience	Glufosinate herbicide tolerance , Antibiotic resistance	bar<br><br><br>nptII *	Streptomyces hygroscopicus<br><br>Escherichia coli Tn5 transposon	phosphinothricin N-acetyltransferase (PAT) enzyme<br>neomycin phosphotransferase II enzyme	eliminates herbicidal activity of glufosinate (Phosphinothricin) herbicides by acetylation<br>Allows transformed plants to metabolize neomycin and kanamycin antibiotics during selection	Australia<br>Canada<br>China<br>European Union<br>Mexico	2002<br>1995<br>2002 *<br>1997 *<br><br>1999	 1995<br>2002 *<br>1998 *	2003<br>1995

							New Zealand<br>South Africa<br>South Korea<br>United States	2002<br>2001<br>2005<br>1995	 2001<br>2008 	 <br><br>2002
HCN92 x MON88302 (ACS-BNøø7-1 x MON-883ø2-9)	Bayer CropScience	Glufosinate herbicide tolerance , Glyphosate herbicide tolerance , Antibiotic resistance	bar<br>nptII *<br>cp4 epsps (aroA:CP4)	Streptomyces hygroscopicus<br>Escherichia coli Tn5 transposon<br>Agrobacterium tumefaciens strain CP4	phosphinothricin N-acetyltransferase (PAT) enzyme<br>neomycin phosphotransferase II enzyme<br>herbicide tolerant form of 5-enolpyruvulshikimate-3-phosphate synthase (EPSPS) enzyme	eliminates herbicidal activity of glufosinate (Phosphinothricin) herbicides by acetylation<br>Allows transformed plants to metabolize neomycin and kanamycin antibiotics during selection<br>decreases binding affinity for glyphosate thereby conferring increased tolerance to glyphosate herbicide	Australia	2016	2016	2016
LBFLFK (BPS-BFLFK-2)	BASF	Modified oil/fatty acid , Imazamox herbicide tolerance	PpD6E<br>TcD5D<br>OtD6D<br>TpD6E<br>PsD12D<br>PirO3D	Physcomitrella patens<br>Thraustochytrium sp.<br>Ostreococcus tauri<br>Thalassiosira pseudonana<br>Phytophthora sojae<br>Pythium irregular	delta-6 elongase<br>two copies of the coding sequence for a delta-5 desaturase cD5D(Tc)1 and cD5D(Tc)2<br>delta-6 desaturase<br>delta-6 elongase<br>delta-12 desaturase<br>two copies of the coding sequence for	catalyzes the decarboxylation Claisen-like condensation of two carbons from malonyl-CoA to C18:3n-6-8-keto-CoA, which is then converted to C20:3n-6-CoA by endogenous enzymes<br><br>converts C18:2n-6 fatty acids into C18:3n-6 fatty acids<br>catalyzes the decarboxylation Claisen-like condensation of two carbons from malonyl-CoA to C18:3n-6-8-keto-CoA, which is then converted to C20:3n-6-CoA by endogenous enzymes<br>convert C18:1n-9 into C18:2n-6<br>converts C20:4n-6 into C20:5n-3	United States			2019

			PiO3D<br>TcD4D<br>PiD4D<br>OtD5E<br>AtAHAS	Phytophthora infestans<br>Thraustochytrium sp.<br>Pavlova Intheri<br>Ostreococcus tauri<br>*Arabidopsis thaliana*	an omega-3 desaturase, cO3D(Pir)1 and cO3D(Pir)2<br>omega-3 desaturase<br>delta-4 desaturase<br>delta-4 desaturase<br>delta-5 elongase<br>Acetohydroxy acid synthase large subunit	converts C20:4n-6 into C20:5n-3<br>converts C22:5n-3 into C22:6n-3<br>converts C22:5n-3 into C22:6n-3<br>catalyzes the decarboxylation Claisen-like condensation of two carbons from malonyl-CoA to C20:5n-3-CoA generating C22:5n-3-8 keto-C CoA, which is then converted to C22:5n-3-CoA by endogenous enzymes<br>Tolerance to the herbicide imazamox				
MON88302(MON-883ø2-9	Monsanto Company	Glyphosate herbicide tolerance	cp4 epsps (aroA:CP4)	*Agrobacterium tumefaciens* strain CP4	herbicide tolerant form of 5-enolpyruvulshikimate-3-phosphate synthase (EPSPS) enzyme	decreases binding affinity for glyphosate thereby conferring increased tolerance to glyphosate herbicide	Australia<br>Canada<br>China<br>European Union<br>Japan<br>Mexico<br>New Zealand<br>Philippines<br>Singapore<br>South Korea<br>United States	2012<br>2012<br>2018 *<br>2015<br>2013<br>2013<br>2012<br>2015<br>2017<br><br>2012	2012<br>2012<br>2018 *<br>2015<br>2013<br><br>2012<br>2015<br><br>2014<br>2012	2014<br>2012<br><br><br>2013<br><br><br><br><br><br>2013
MON88302x MS8 x RF3 (MON-883ø2-9 x ACS-BNøø5-8 x ACS-BNøø3-6	Monsanto Company	Glufosinate herbicide tolerance , Glyphosate herbicide tolerance , Male sterility , Fertility	cp4 epsps (aroA:CP4)<br>bar	*Agrobacterium tumefaciens strain CP4*<br>*Streptomyces hygroscopicus*	herbicide tolerant form of 5-enolpyruvulshikimate-3-phosphate synthase (EPSPS) enzyme<br>phosphinothricin N-acetyltransferase (PAT) enzyme<br>barnase	decreases binding affinity for glyphosate thereby conferring increased tolerance to glyphosate herbicide<br>eliminates herbicidal activity of glufosinate (Phosphinothricin) herbicides by acetylation<br>causes male sterility by interfering with	European Union<br>Japan<br>Mexico<br>South Korea<br>Taiwan	2017<br>2015<br>2015<br>2014<br>2017	2017<br>2015<br><br>2015	 2015 *

		restoration	barnase<br><br>barstar	*Bacillus amyloliquefaciens*<br>*Bacillus amyloliquefaciens*	ribonuclease(RNAse) enzyme<br>barnase ribonuclease inhibitor	RNA production in the tapetum cells of the anther<br>restores fertility by repressing the inhibitory effect of barnase on tapetum cells of the anther				
MON88302x RF3 (MON-883ø2-9 x ACS-BNøø3-6	Bayer CropScience	Glufosinate herbicide tolerance, Glyphosate herbicide tolerance, Fertility restoration	cp4 epsps (aroA:CP4)<br><br>bar<br><br>barstar	Agrobacterium tumefaciens strain CP4<br><br>Streptomyces hygroscopicus<br><br>Bacillus amyloliquefaciens	herbicide tolerant form of 5-enolpyruvulshikimate-3-phosphate synthase (EPSPS) enzyme<br>phosphinothricin N-acetyltransferase (PAT) enzyme<br>barnase ribonuclease inhibitor	decreases binding affinity for glyphosate thereby conferring increased tolerance to glyphosate herbicide<br><br>eliminates herbicidal activity of glufosinate (Phosphinothricin) herbicides by acetylation<br>restores fertility by repressing the inhibitory effect of barnase on tapetum cells of the anther	Australia<br>Japan<br>Mexico<br>South Korea<br>Taiwan	2016<br>2015<br>2015<br>2014<br><br>2017	2015<br>2015	2016<br>2015 *
MPS961 (not available)	BASF	Phytase production, Antibiotic resistance	nptII *<br><br>phyA	Escherichia coli Tn5 transposon<br><br>Aspergillus niger var. Van Tieghem	neomycin phosphotransferase II enzyme<br>3-phytase enzyme	allows transformed plants to metabolize neomycin and kanamycin antibiotics during selection<br>increases the breakdown of plant phytates which bind phosphorus and makes the latter available to monogastric animals	United States	1999	1999	
MPS962 (not available	BASF	Phytase production , Antibiotic resistance	nptII *<br><br>phyA	Escherichia coli Tn5 transposon<br><br>Aspergillus niger var. Van Tieghem	neomycin phosphotransferase II enzyme<br>3-phytase enzyme	allows transformed plants to metabolize neomycin and kanamycin antibiotics during selection<br>increases the breakdown of plant phytates which bind phosphorus and makes the latter available to monogastric animals	United States	1999	1999	
MPS963 (not available	BASF	Phytase production , Antibiotic resistance	nptII *<br><br>phyA	Escherichia coli Tn5 transposon<br><br>Aspergillus niger var. Van Tieghem	neomycin phosphotransferase II enzyme<br>3-phytase enzyme	allows transformed plants to metabolize neomycin and kanamycin antibiotics during selection<br>increases the breakdown of plant phytates which bind phosphorus and makes the latter available to monogastric animals	United States	1999	1999	1998
MPS964 (not available	BASF	Phytase production , Antibiotic resistance	nptII *<br><br>phyA	Escherichia coli Tn5 transposon<br><br>Aspergillus niger	neomycin phosphotransferase II enzyme<br>3-phytase enzyme	allows transformed plants to metabolize neomycin and kanamycin antibiotics during selection<br>increases the breakdown of plant	United States	1999	1999	

				var. Van Tieghem		phytates which bind phosphorus and makes the latter available to monogastric animals				
MPS965 (not available	BASF	Phytase production , Antibiotic resistance	nptII *<br>phyA	Escherichia coli Tn5 transposon<br>Aspergillus niger var. Van Tieghem	neomycin phosphotransferase II enzyme<br>3-phytase enzyme	allows transformed plants to metabolize neomycin and kanamycin antibiotics during selection<br>increases the breakdown of plant phytates which bind phosphorus and makes the latter available to monogastric animals	United States	1999	1999	
MS1 (B91-4) (ACS-BNøø4-7)	Bayer CropScience	Glufosinate herbicide tolerance , Male sterility , Antibiotic resistance	bar<br>barnase<br>nptII *	Streptomyces hygroscopicus<br>Bacillus amyloliquefaciens<br>Escherichia coli Tn5 transposon	phosphinothricin N-acetyltransferase (PAT) enzyme<br>barnase ribonuclease(RNAse) enzyme<br>neomycin phosphotransferase II enzyme	eliminates herbicidal activity of glufosinate (Phosphinothricin) herbicides by acetylation<br>causes male sterility by interfering with RNA production in the tapetum cells of the anther<br>allows transformed plants to metabolize neomycin and kanamycin antibiotics during selection	Australia<br>Canada<br>China<br>European Union<br>Mexico<br>New Zealand<br>United States	2002<br>1995<br>2002 *<br>1997 *<br>1999<br>2002<br>1996	 1995<br>2002 *<br>1996 *<br><br><br>1996	2003<br>1995<br><br><br><br><br>2002
MS1 x MON88302 (ACS- BNøø4-7 x MON- 883ø2-9)	Bayer CropScience	Glufosinate herbicide tolerance , Glyphosate herbicide tolerance , Male sterility , Antibiotic resistance	cp4 epsps (aroA:CP4)<br>bar<br>barnase<br>nptII *	Agrobacterium tumefaciens strain CP4<br>Streptomyces hygroscopicus<br>Bacillus amyloliquefaciens<br>Escherichia coli Tn5 transposon	herbicide tolerant form of 5-enolpyruvulshikimate-3-phosphate synthase (EPSPS) enzyme<br>phosphinothricin N-acetyltransferase (PAT) enzyme<br>barnase ribonuclease(RNAse) enzyme<br>neomycin phosphotransferase II enzyme	decreases binding affinity for glyphosate thereby conferring increased tolerance to glyphosate herbicide<br>eliminates herbicidal activity of glufosinate (Phosphinothricin) herbicides by acetylation<br>causes male sterility by interfering with RNA production in the tapetum cells of the anther<br>allows transformed plants to metabolize neomycin and kanamycin antibiotics during selection	Australia	2016	2016	2016
MS1 x RF1 (PGS1) (ACS- BNøø4-7 x ACS- BNøø1-4)	BASF	Glufosinate herbicide tolerance , Male sterility , Fertility restoration ,	bar<br>barnase<br>barstar	Streptomyces hygroscopicus<br>Bacillus amyloliquefaciens<br>Bacillus	phosphinothricin N-acetyltransferase (PAT) enzyme<br>barnase ribonuclease(RNAse) enzyme<br>barnase ribonuclease	eliminates herbicidal activity of glufosinate (Phosphinothricin) herbicides by acetylation<br>causes male sterility by interfering with RNA production in the tapetum cells of the anther<br>restores fertility by repressing the	Australia<br>Canada<br>China<br>European Union<br>Japan<br>Mexico	2002<br>1994<br>2002 *<br>1997 *<br>2001<br>1999	 1995<br>2002 *<br>1996 *<br>2003	2003<br>1995

		Antibiotic resistance	nptII *	amyloliquefaciens<br>Escherichia coli Tn5 transposon	inhibitor<br>neomycin phosphotransferase II enzyme	inhibitory effect of barnase on tapetum cells of the anther<br>allows transformed plants to metabolize neomycin and kanamycin antibiotics during selection	New Zealand<br>South Africa<br>South Korea	2002<br>2001<br>2005	 2001<br>2008	
MS1 x RF2 (PGS2) (ACS- BNøø4-7 x ACS- BNøø2-5)	BASF	Glufosinate herbicide tolerance , Male sterility , Fertility restoration , Antibiotic resistance	bar<br>barnase<br>barstar<br>nptII *	Streptomyces hygroscopicus<br>Bacillus amyloliquefaciens<br>Bacillus amyloliquefaciens<br>Escherichia coli Tn5 transposon	phosphinothricin N-acetyltransferase (PAT) enzyme<br>barnase ribonuclease(RNAse) enzyme<br>barnase ribonuclease inhibitor<br>neomycin phosphotransferase II enzyme	eliminates herbicidal activity of glufosinate (Phosphinothricin) herbicides by acetylation<br>causes male sterility by interfering with RNA production in the tapetum cells of the anther<br>restores fertility by repressing the inhibitory effect of barnase on tapetum cells of the anther<br>allows transformed plants to metabolize neomycin and kanamycin antibiotics during selection	Australia<br>Canada<br>China<br>European Union<br>Japan<br>New Zealand<br>South Africa<br>South Korea	2002<br>1995<br>2002 *<br>1997 *<br>2001<br>2002<br>2001<br>2005	 1995<br>2002 *<br>1997 *<br>2003<br><br>2001<br>2008	2003<br>1995
MS1 x RF3 (ACS-BNøø4-7 x ACS-BNøø3-6)	Bayer CropScience	Glufosinate herbicide tolerance , Male sterility , Fertility restoration , Antibiotic resistance	bar<br>barnase<br>barstar<br>nptII *	Streptomyces hygroscopicus<br>Bacillus amyloliquefaciens<br>Bacillus amyloliquefaciens<br>Escherichia coli Tn5 transposon	phosphinothricin N-acetyltransferase (PAT) enzyme<br>barnase ribonuclease(RNAse) enzyme<br>barnase ribonuclease inhibitor<br>neomycin phosphotransferase II enzyme	eliminates herbicidal activity of glufosinate (Phosphinothricin) herbicides by acetylation<br>causes male sterility by interfering with RNA production in the tapetum cells of the anther<br>restores fertility by repressing the inhibitory effect of barnase on tapetum cells of the anther<br>allows transformed plants to metabolize neomycin and kanamycin antibiotics during selection	China	2006 *	2006 *	
MS11 (BCS-BNø12-7)	Bayer CropScience	Glufosinate herbicide tolerance , Male sterility , Fertility restoration	barnase<br>bar<br>barstar	Bacillus amyloliquefaciens<br>Streptomyces hygroscopicus<br>Bacillus amyloliquefaciens	barnase ribonuclease(RNAse) enzyme<br>phosphinothricin N-acetyltransferase (PAT) enzyme<br>barnase ribonuclease inhibitor	causes male sterility by interfering with RNA production in the tapetum cells of the anther<br>eliminates herbicidal activity of glufosinate (Phosphinothricin) herbicides by acetylation<br>restores fertility by repressing the inhibitory effect of barnase on tapetum cells of the anther	Australia<br>Canada<br>New Zealand<br>Philippines<br>South Korea<br>Taiwan<br>United States	2017<br>2018<br>2017<br>2019 *<br>2019 *<br>2018<br>2017	 2018<br><br>2019 *<br><br><br>2017	 2018<br><br><br><br><br>2017
MS8 (ACS-	Bayer	Glufosinate	bar	Streptomyces	phosphinothricin N-	eliminates herbicidal activity of	Australia	2002		2003

BNøø5-8)	CropScience	herbicide tolerance , Male sterility	 <br>barnase	hygroscopicus<br><br>Bacillus amyloliquefaciens	acetyltransferase (PAT) enzyme<br>barnase ribonuclease(RNAse) enzyme	glufosinate (Phosphinothricin) herbicides by acetylation<br>causes male sterility by interfering with RNA production in the tapetum cells of the anther	Canada<br>European Union<br>Japan<br>New Zealand<br>Philippines<br>South Korea<br>Taiwan<br>United States	1997<br>2013 *<br>2001<br>2002<br><br>2018 *<br>2013<br><br>2015<br>1998	1996<br>2007 *<br><br>2003<br><br><br>2018 *<br>2012<br><br><br>1998	1996<br><br><br>2006<br><br><br><br><br><br>1999
MS8 x MON88302(ACS-BNøø5-8 x MON-883ø2-9)	Bayer CropScience	Glufosinate herbicide tolerance , Glyphosate herbicide tolerance , Male sterility	cp4 epsps (aroA:CP4)<br><br>bar<br><br>barnase	Agrobacterium tumefaciens strain CP4<br><br>Streptomyces hygroscopicus<br><br>Bacillus amyloliquefaciens	herbicide tolerant form of 5-enolpyruvulshikimate-3-phosphate synthase (EPSPS) enzyme<br>phosphinothricin N-acetyltransferase (PAT) enzyme<br>barnase ribonuclease(RNAse) enzyme	decreases binding affinity for glyphosate thereby conferring increased tolerance to glyphosate herbicide<br><br>eliminates herbicidal activity of glufosinate (Phosphinothricin) herbicides by acetylation<br>causes male sterility by interfering with RNA production in the tapetum cells of the anther	Australia	2016	2016	2016
MS8 x RF3 (ACS-BNøø5-8 x ACS-BNøø3-6)	Bayer CropScience	Glufosinate herbicide tolerance, Male sterility, Fertility restoration	bar<br><br>barnase<br><br>barstar	Streptomyces hygroscopicus<br><br>Bacillus amyloliquefaciens<br><br>Bacillus amyloliquefaciens	phosphinothricin N-acetyltransferase (PAT) enzyme<br>barnase ribonuclease(RNAse) enzyme<br>barnase ribonuclease inhibitor	eliminates herbicidal activity of glufosinate (Phosphinothricin) herbicides by acetylation<br>causes male sterility by interfering with RNA production in the tapetum cells of the anther<br>restores fertility by repressing the inhibitory effect of barnase on tapetum cells of the anther	Australia<br>Canada<br>China<br>European Union<br>Japan<br>Malasia<br>Mexico<br>New Zealand<br>South Africa<br>South Korea<br>Taiwan	2002<br>1997<br>2002 *<br>2013 *<br><br>2001<br>2016<br>2004<br>2002<br><br>2001<br><br>2005<br><br>2015	 1996<br>2002 *<br>2007 *<br><br>2003<br>2016<br><br><br><br>2001<br><br>2005	2003<br>1996<br><br><br><br>2007
MS8 x RF3 x GT73 (RT73) (ACS-BNøø5-8 x ACS-	Bayer CropScience	Glufosinate herbicide tolerance,	bar	Streptomyces hygroscopicus	phosphinothricin N-acetyltransferase (PAT) enzyme	eliminates herbicidal activity of glufosinate (Phosphinothricin) herbicides by acetylation	Japan<br>Mexico<br>South	2011<br>2011<br>2015	2010<br><br>2015	2012

BNøø3-6 x MON-øøø73-7)<br><br>OXY-235 (ACS-BNø11-5)	Bayer CropScience	Glyphosate herbicide tolerance<br>Male sterility, Fertility restoration<br><br>Oxynil herbicide tolerance	barnase<br>barstar<br>cp4 epsps (aroA:CP4<br>goxv247<br>bxn	Bacillus amyloliquefaciens<br>Bacillus amyloliquefaciens<br>Agrobacterium tumefaciens strain CP4<br>Ochrobactrum anthropi strain LBAA<br>Klebsiella pneumonia subsp. Ozaenae	barnase ribonuclease(RNAse) enzyme<br>barnase ribonuclease inhibitor<br>herbicide tolerant form of 5-enolpyruvulshikimate-3-phosphate synthase (EPSPS) enzyme<br>glyphosate oxidase<br>nitrilase enzyme	causes male sterility by interfering with RNA production in the tapetum cells of the anther<br>restores fertility by repressing the inhibitory effect of barnase on tapetum cells of the anther<br>decreases binding affinity for glyphosate thereby conferring increased tolerance to glyphosate herbicide<br>confers tolerance to glyphosate herbicides by degrading glyphosate into aminomethylphosphonic acid (AMPA) and glyoxylate<br>eliminates herbicidal activity of oxynil herbicides (eg. Bromoxynil)	Korea<br>Taiwan<br>Australia<br>Canada<br>China<br>Japan<br>New Zealand<br>United States	2016<br>2002<br>1997<br>2002 *<br>2001<br>2002<br>1999	1997<br>2002 *<br>2003	1997<br>2008
PHY14 (not available)	Bayer CropScience	Glufosinate herbicide tolerance, Male sterility, Fertility restoration	bar<br>barnase<br>barstar	Streptomyces hygroscopicus<br>Bacillus amyloliquefaciens<br>Bacillus amyloliquefaciens	phosphinothricin N-acetyltransferase (PAT) enzyme<br>barnase ribonuclease(RNAse) enzyme<br>barnase ribonuclease inhibitor	eliminates herbicidal activity of glufosinate (Phosphinothricin) herbicides by acetylation<br>causes male sterility by interfering with RNA production in the tapetum cells of the anther<br>restores fertility by repressing the inhibitory effect of barnase on tapetum cells of the anther	No appovals found			
PHY23 (not available)	Bayer CropScience	Glufosinate herbicide tolerance, Male sterility Fertility restoration	bar<br>barnase<br>barstar	Streptomyces hygroscopicus<br>Bacillus amyloliquefaciens<br>Bacillus amyloliquefaciens	phosphinothricin N-acetyltransferase (PAT) enzyme<br>barnase ribonuclease(RNAse) enzyme<br>barnase ribonuclease inhibitor	eliminates herbicidal activity of glufosinate (Phosphinothricin) herbicides by acetylation<br>causes male sterility by interfering with RNA production in the tapetum cells of the anther<br>restores fertility by repressing the inhibitory effect of barnase on tapetum cells of the anther	No appovals found			
PHY35 (not available)	Bayer CropScience	Glufosinate herbicide tolerance, Male sterility, Fertility restoration	bar<br>barnase<br>barstar	Streptomyces hygroscopicus<br>Bacillus amyloliquefaciens<br>Bacillus	phosphinothricin N-acetyltransferase (PAT) enzyme<br>barnase ribonuclease(RNAse) enzyme<br>barnase ribonuclease	eliminates herbicidal activity of glufosinate (Phosphinothricin) herbicides by acetylation<br>causes male sterility by interfering with RNA production in the tapetum cells of the anther<br>restores fertility by repressing the	No appovals found			

				amyloliquefaciens	inhibitor	inhibitory effect of barnase on tapetum cells of the anther				
PHY36 (not available)	Bayer CropScience	Glufosinate herbicide tolerance, Male sterility, Fertility restoration	bar<br><br>barnase<br><br>barstar	Streptomyces hygroscopicus<br><br>Bacillus amyloliquefaciens<br><br>Bacillus amyloliquefaciens	phosphinothricin N-acetyltransferase (PAT) enzyme<br>barnase ribonuclease(RNAse) enzyme<br>barnase ribonuclease inhibitor	eliminates herbicidal activity of glufosinate (Phosphinothricin) herbicides by acetylation<br>causes male sterility by interfering with RNA production in the tapetum cells of the anther<br>restores fertility by repressing the inhibitory effect of barnase on tapetum cells of the anther	No appovals found			
RF1 (B93-101) (ACS- BNøø1-4)	Bayer CropScience	Glufosinate herbicide tolerance, Fertility restoration, Antibiotic resistance	bar<br><br>barstar<br><br>nptII *	Streptomyces hygroscopicus<br><br>Bacillus amyloliquefaciens<br><br>Escherichia coli Tn5 transposon	phosphinothricin N-acetyltransferase (PAT) enzyme enzyme<br>barnase ribonuclease inhibitor<br><br>neomycin phosphotransferase II enzyme	eliminates herbicidal activity of glufosinate (Phosphinothricin) herbicides by acetylation<br><br>restores fertility by repressing the inhibitory effect of barnase on tapetum cells of the anther<br>allows transformed plants to metabolize neomycin and kanamycin antibiotics during selection	Australia<br>Canada<br>China<br>European Union<br>Mexico<br>New Zealand<br>United States	2002<br>1994<br>2002 *<br>1997 *<br><br>1999<br>2002<br><br>1996	 199502002<br>*<br>1996 *<br><br><br><br><br>1996	2003<br>1995<br><br><br><br><br><br><br>2002
RF1 x MON88302 (ACS- BNøø1-4 x MON- 883ø2-9)	Bayer CropScience	Glufosinate herbicide tolerance, Glyphosate herbicide tolerance, Fertility restoration, Antibiotic resistance	cp4 epsps (aroA:CP4)<br><br>bar<br><br>barstar<br><br>nptII *	Agrobacterium tumefaciens strain CP4<br><br>Streptomyces hygroscopicus<br><br>Bacillus amyloliquefaciens<br><br>Escherichia coli Tn5 transposon	herbicide tolerant form of 5-enolpyruvulshikimate-3-phosphate synthase (EPSPS) enzyme<br>phosphinothricin N-acetyltransferase (PAT) enzyme<br>barnase ribonuclease inhibitor<br>neomycin phosphotransferase II enzyme	decreases binding affinity for glyphosate thereby conferring increased tolerance to glyphosate herbicide<br><br>eliminates herbicidal activity of glufosinate (Phosphinothricin) herbicides by acetylation<br>restores fertility by repressing the inhibitory effect of barnase on tapetum cells of the anther<br>allows transformed plants to metabolize neomycin and kanamycin antibiotics during selection	Australia	2016	2016	2016
RF2 (B94-2) (ACS-BNøø2-5)	Bayer CropScience	Glufosinate herbicide tolerance , Fertility restoration , Antibiotic	bar<br><br>barstar	Streptomyces hygroscopicus<br><br>Bacillus amyloliquefaciens	phosphinothricin N-acetyltransferase (PAT) enzyme enzyme<br>barnase ribonuclease inhibitor	eliminates herbicidal activity of glufosinate (Phosphinothricin) herbicides by acetylation<br><br>restores fertility by repressing the inhibitory effect of barnase on tapetum	Australia<br>Canada<br>China<br>European Union<br>New	2002<br>1995<br>2002 *<br>1997 *<br><br>2002	 1995<br>2002 *<br>1997 *	2003<br>1995

		resistance	 nptII *	 Escherichia coli<br>Tn5 transposon	 neomycin<br>phosphotransferase II<br>enzyme	cells of the anther<br>allows transformed plants to metabolize<br>neomycin and kanamycin antibiotics<br>during selection	Zealand<br>United<br>States	 1996	 1996	 2002
RF2 x MON88302<br>(ACS- BNøø2-5 x<br>MON- 883ø2-9)	Bayer<br>CropScience	Glufosinate<br>herbicide<br>tolerance,<br>Glyphosate<br>herbicide<br>tolerance,<br>Fertility<br>restoration,<br>Antibiotic<br>resistance	cp4 epsps<br>(aroA:CP4)<br><br><br>bar<br><br><br>barstar<br><br><br>nptII *	Agrobacterium<br>tumefaciens strain<br>CP4<br><br>Streptomyces<br>hygroscopicus<br><br>Bacillus<br>amyloliquefaciens<br><br>Escherichia coli<br>Tn5 transposon	herbicide tolerant<br>form of 5-<br>enolpyruvulshikimate-<br>3-phosphate synthase<br>(EPSPS) enzyme<br>phosphinothricin N-<br>acetyltransferase<br>(PAT) enzyme<br>barnase ribonuclease<br>inhibitor<br>neomycin<br>phosphotransferase II<br>enzyme	decreases binding affinity for<br>glyphosate thereby conferring increased<br>tolerance to glyphosate herbicide<br><br>eliminates herbicidal activity of<br>glufosinate (Phosphinothricin)<br>herbicides by acetylation<br>restores fertility by repressing the<br>inhibitory effect of barnase on tapetum<br>cells of the anther<br>allows transformed plants to metabolize<br>neomycin and kanamycin antibiotics<br>during selection	Australia	2016	2016	2016
RF3 (ACS- BNøø3-<br>6)	BASF	Glufosinate<br>herbicide<br>tolerance,<br>Fertility<br>restoration	bar<br><br><br><br>barstar	Streptomyces<br>hygroscopicus<br><br><br>Bacillus<br>amyloliquefaciens	phosphinothricin N-<br>acetyltransferase<br>(PAT) enzyme<br>enzyme<br>barnase ribonuclease<br>inhibitor	eliminates herbicidal activity of<br>glufosinate (Phosphinothricin)<br>herbicides by acetylation<br><br>restores fertility by repressing the<br>inhibitory effect of barnase on tapetum<br>cells of the anther	Australia<br>Canada<br>China<br>European<br>Union<br>Japan<br>Mexico<br>New<br>Zealand<br>Philippines<br>South<br>Korea<br>Taiwan<br>United<br>States	2002<br>1997<br>2018 *<br><br>2013 *<br><br><br>2007<br>2002<br>2018<br>2013<br>2015<br>1998	 1996<br>2018 *<br>2017 *<br><br>2003<br><br><br><br><br>2018<br>2012<br><br>1998	2003<br>1996<br><br><br><br>2007 *<br><br><br><br><br><br><br><br>1999
